# Definition and Classification of Dental Hygiene Interventions Based on the Dental Hygiene Process of Care: A Delphi Study

**DOI:** 10.3390/ijerph20176704

**Published:** 2023-09-02

**Authors:** Seon-Hui Kwak, Soo-Myoung Bae, Sun-Jung Shin, Bo-Mi Shin

**Affiliations:** 1 Department of Dental Hygiene, College of Dentistry, Gangneung Wonju National University, Gangneung-si 25457, Republic of Korea; tjsgml0617@hanmail.net (S.-H.K.); edelweiss@gwnu.ac.kr (S.-M.B.); freshjung@gwnu.ac.kr (S.-J.S.); 2 Research Institute of Dental Hygiene Science, Gangneung Wonju National University, Gangneung-si 25457, Republic of Korea; 3 Research Institute of Oral Science, Gangneung Wonju National University, Gangneung-si 25457, Republic of Korea

**Keywords:** dental hygiene intervention, dental hygiene practice, dental hygiene process of care, dental hygienist

## Abstract

This study aimed to conceptualize the dental hygiene intervention performed by dental hygienists based on the dental hygiene process of care. The dental hygiene intervention classification was conducted on the qualitative content analysis method. The contents of the primary dental hygiene intervention classification were refined after review by three internal experts. The final classification of dental hygiene interventions was derived through an expert Delphi survey conducted twice with 15 professors in charge of clinical dental hygiene. In the Delphi survey, the content validity and clarity were evaluated. As a result of the first and second expert Delphi surveys, the content validity ratio for all dental hygiene interventions and definitions was ≥0.60, and the content validity index was ≥0.80. The degree of agreement was a minimum of 0.80 and a maximum of 1.00. Thirty-eight dental hygiene interventions were conceptualized, and the essence of the dental hygienist was confirmed. Dental hygienists are oral health experts who help in preventing oral diseases and promoting oral health by providing evidence-based comprehensive preventive management through interaction with their clients, and they are a primary care worker who could contribute to health promotion. In the future, dental hygienists are expected to be actively involved in the primary care system and dental clinical sites and contribute to health promotion through practical discussions for this purpose.

## 1. Introduction

According to the International Federation of Dental Hygienists (IFDH), a dental hygienist is a primary oral healthcare professional who provides general and oral healthcare throughout a patient’s life [1]. Worldwide, dental hygienists have served as clinicians, administrators, communicators, collaborators, critical thinkers, advocates, and coordinators who provide patient-centered and prevention-centered comprehensive oral care to promote personal and social health and achieve health equity [2,3]. The role of these dental hygienists was further emphasized in the World Health Organization’s (WHO) “Draft Global Strategy on Oral Health” announced in 2021 [4]. According to a WHO report, the prevalence of dental caries, periodontal disease, and tooth loss has remained higher than the prevalence of other chronic diseases over the past 30 years. Accordingly, the WHO suggested that healthcare providers must offer preventive oral care centered on primary care, with an emphasis on addressing common risk factors related to oral health. Furthermore, it is worth noting that the current medical system often prioritizes the therapeutic approach. In light of this limitation, the World Health Organization (WHO) has recommended that dental hygienists, who specialize in oral health promotion and disease prevention, can play a crucial role in developing preventive strategies and facilitating primary care [4].

Dental hygienists aim to solve the oral health problems of clients and local residents based on a standard of practice called the dental hygiene process of care to promote oral health, prevent oral diseases, and provide oral care to satisfy unmet human needs [2,3]. The American [5] and Canadian [6] Dental Hygienists Associations define the dental hygiene process of care as follows: “To provide a framework where the individualized needs of the patient can be met; and to identify the causative or influencing factors of a condition that can be reduced, eliminated, or prevented by the dental hygienist”. Dental hygiene care is not a novel medical treatment technique. It encompasses various clinical practices, such as examinations, preventive treatments, and oral health education that fall within the scope of practice of dental hygienists. These practices are conducted following a systematic framework that includes assessment, diagnosis, planning, implementation, evaluation, and documentation [7]. The evolution of the dental hygiene process of care has served as the foundation for the progression of dental hygienists’ role. They have transitioned from being assistants who aided dentists in treatment to becoming professionals who provide healthcare within the scope of dental hygiene practice. This transformation is based on their responsibility for critical thinking and decision making [7].

As the American Dental Hygienist Association (ADHA) announced the dental hygiene process of care as the standard for dental hygiene practice in 1985, the dental hygiene science has quantitatively and qualitatively evolved to establish the professionalism of dental hygienists and develop their capabilities. The Commission on Dental Accreditation (CODA) included the contents of the dental hygiene process of care in the evaluation items for accreditation of dental hygiene educational institutions, and it stipulated that the dental hygiene process of care could be used in various ways in the dental hygiene curriculum [8]. Consequently, dental hygiene departments around the world have introduced an educational system based on the dental hygiene process of care. For example, at the University of Bridgeport in the United States, the bachelor’s degree in dental hygiene operated a process of care curriculum in compliance with the American Dental Education Association Competencies for Entry into the Profession of Dental Hygiene. To strengthen dental hygienists’ clinical competence, the program offers theoretical and practical classes ranging from dental hygiene assessment to evaluation [9]. In Korea, the dental hygiene process of care was introduced in earnest in 2005, starting with Lee and Cho’s [10] theoretical review study on the dental hygiene process. Additionally, the dental hygiene process of care curriculum, which borrowed the American dental hygiene education curriculum and considered the scope of dental hygienists’ work in Korea, began to be operated centered on the bachelor’s degree in dental hygiene.

In addition, the American and Canadian Dental Hygienists Associations developed and distributed standard guidelines for clinical dental hygiene practice [5,6]. Additionally, Korea has also developed practical guidelines called the “Standards for Clinical Dental Hygiene Practice in Korea” [11]. The practical guidelines included specific guidelines for dental hygienists to provide optimal dental hygiene management through critical thinking and evidence-based decision making at each stage of the dental hygiene process of care. Furthermore, the guidelines contributed to the standardization of dental hygiene curricula. Additionally, in Korea, a job description was developed to articulate the role of a dental hygienist and to capture the scope of practice [12]. Bae et al. [13] suggested detailed dental hygiene core competencies based on the certification evaluation criteria for dental hygiene education presented by CODA. However, as previously developed practical guidelines and previous studies have focused on the techniques and equipment required for the dental hygiene process of care, limitations remain in expressing the originality and professionalism of dental hygiene practice.

In the field of nursing science, which holds similarities to dental hygiene science, the duties performed by nurses are classified and conceptualized as “nursing interventions” that uphold the nature, expertise, and originality of nurses [14]. Classification refers to the process of forming a concept by organizing and naming groups that share similar characteristics [15]. The University of Iowa research team named the unique tasks performed by nurses in clinical practice through standardized terms called the Nursing Intervention Classification (NIC), and by evaluating the effectiveness of nursing by building a database of standardized nursing terms, they contributed to describing the nature, originality, professionalism, and socioeconomic value of nursing services [16,17,18,19]. The NIC systematically organized the nursing practices to improve the health of patients based on the nurse’s clinical judgment, and it describes “care” as the essence and expertise of nurses. The NIC describes the “care” that nurses provide in all practice settings, including physiological and psychosocial domains, disease treatment and prevention, health promotion, interventions for individuals, care, and communities, and indirect care, and published 565 nursing interventions in 2013 [14].

Recent studies that explain the practices and roles of dental hygienists and have standardized the dental hygiene process of care have been actively conducted worldwide [5,6,11,13]. Professions have systematic theories, specialized knowledge and skills to perform tasks, and have a monopoly on labor [20]. While dental hygienists were recognized as professionals who provided services to prevent oral disease and promote oral health, systematic and specific conceptualized research on the unique practices that dental hygienists perform as professionals was lacking. Therefore, this study aimed to conceptualize and systematize the professional activities of dental hygienists by classifying and defining the unique practices performed by dental hygienists based on the dental hygiene process of care through dental hygiene interventions. 

## 2. Materials and Methods

### 2.1. Establishing Operational Definitions for Dental Hygiene Intervention

This study established operational definitions of dental hygiene interventions by comparing those of nursing interventions [21]. The researcher reviewed domestic and foreign precedent literature using keywords such as “dental hygienist”, “practices of dental hygienists”, and “dental hygiene process of care”. Based on the reviewed content, it was defined to express the essence and value of dental hygienists and the professionalism of dental hygienist work. As such, this study operationally defined “dental hygiene intervention” as “a treatment performed by a dental hygienist based on the judgment and knowledge of the client’s health status to satisfy the client’s dental hygiene’s human needs, prevent oral diseases, and improve oral and general health”.

### 2.2. Writing Principles for Dental Hygiene Intervention Classification 

This study established a standardized language system for dental hygiene intervention classification to establish the “Principles for Writing Dental Hygiene Intervention Classification”. The Writing Principles were adapted from the NIC while considering the academic and clinical characteristics of dental hygiene science [22].

### 2.3. Qualitative Content Analysis for the Classification of Dental Hygiene Interventions

To analyze dental hygiene interventions, a qualitative content analysis was conducted based on previous literature related to the dental hygiene process of care [23]. Qualitative content analysis was a research method that could be used to identify the implicit meaning and intention contained in document data such as statistical data, diaries, letters, books, medical records, reports, and memoirs [24,25].

This study was conducted in three stages: preparation, organization, and reporting, by applying the qualitative content analysis proposed by Elo and Kyngas [26]. First, the population, approach, and unit of analysis were established in the preparation stage. The population was selected based on previous literature, such as domestic and foreign dental hygiene textbooks, theses, and reports that contained the contents of practice performed by dental hygienists based on the dental hygiene process of care. As for the approach, the “deductive approach” was chosen to conceptualize the activities of dental hygienists comprehensively introduced more specifically in previous studies. The unit of analysis was set as a sentence representing interventional activities performed by dental hygienists, based on the dental hygiene process of care.

At the organizational level, researchers have developed a categorization matrix for dental hygiene interventions based on the practice guidelines articulated in the “Standards for Clinical Dental Hygiene Practice in Korea”. We repeatedly read the contents, conceptual meanings, clinical significance, application methods, and examples of applications contained in the practice guidelines and summarized the keywords included in the text. Then, after grouping keywords with similar meanings, higher-level words that can encompass these words were created. At this time, the higher-level word was “dental hygiene intervention”.

Finally, in the reporting stage, dental hygiene interventions and definitions derived according to the dental hygiene intervention classification writing framework were recorded. The third phase of qualitative content analysis resulted in 65 draft dental hygiene interventions.

### 2.4. Validity Evaluation of Dental Hygiene Intervention Classification 

#### 2.4.1. Inter-Researcher Review

Four researchers with rich experience in research and education on the dental hygiene process of care reviewed and refined the dental hygiene interventions and definitions derived through qualitative content analysis. The inter-researcher review was conducted twice. The first review focused on the aforementioned draft dental hygiene interventions. Compliance with the writing principles of dental hygiene interventions, similarities between items, and validity were reviewed. A total of 46 dental hygiene intervention lists were derived by revising and supplementing the dental hygiene intervention matrix in the first inter-researcher review. The second review focused on the definition of the 46 dental hygiene interventions. The researchers repeatedly read the definitions and reviewed compliance with writing principles, appropriateness of words and context, and validity of the contents.

#### 2.4.2. Expert Delphi Survey

1. Selection of Subjects for Expert Delphi Survey

To evaluate the validity of the dental hygiene intervention classification, from 25 March to 3 May 2022, an expert Delphi survey was conducted twice among 15 dental hygiene department professors who agreed to participate in the study. The expert Delphi survey subjects were selected via convenience sampling from those who had more than five years of experience in teaching the dental hygiene process of care in the dental hygiene department and were dental hygienists with experience in researching the subject of dental hygienist practices, clinical dental hygiene, and dental hygiene process of care.

2. Development of Expert Delphi Survey Questionnaire

A semi-structured questionnaire was developed that consisted of closed-ended questions to evaluate the validity of the 46 dental hygiene intervention classifications derived from the inter-researcher review, and open-ended questions to prepare the expert panel’s opinions on corrections and supplements. The contents of the questionnaire were developed to be evaluated on a five-point Likert scale (1 = not valid at all to 5 = very valid) for “content validity” and “clarity”, as suggested by McCloskey and Bulechek [27]. The items of the expert Delphi survey are listed in Table 1.

3. Analysis of the Expert Delphi Survey Results

Content validity was analyzed by subdividing it into a content validity ratio (CVR), a content validity index (CVI), the degree of convergence, and the degree of agreement. Content validity was evaluated using the content validity ratio (CVR) proposed by Lawshe [28] and the content validity index (CVI) proposed by Lynn [29]. CVR was evaluated by an expert on the validity of how relevant an item is to the research topic and was calculated using the formula CVR = (n−0.5N)/(0.5N). At this time, “n” represents the number of panels who answered 4 or 5 points, and “N” represents the total number of panels who participated in the survey. According to the CVR threshold criterion proposed by Lawshe [28], this study considered content validity to be satisfied when the CVR value was 0.49 or higher. CVI is a method for calculating the percentage of experts who answered “very valid” (5 points) and “reasonable” (4 points) after asking a panel of experts to answer whether the question was related to the research topic. According to the CVI threshold criterion proposed by Lynn [29], when the value was 0.78 or higher, the content validity of the item was evaluated as being satisfied.

The degree of agreement between the panels participating in the Delphi survey could be analyzed through the “degree of convergence” and the “degree of agreement” [30]. The degree of convergence is an index representing the degree of convergence of the panel response results obtained through the Delphi survey and has a value of 0 when all opinions converge at one point. The larger the difference in opinion between the panels, the larger the value. The degree of agreement was a method of verifying how much consensus the opinions of the panel had reached, and it had a value of 1 when there was complete agreement and the value decreased when there was a large disagreement. Additionally, the researcher confirmed the coefficient of variation (CV) to objectively measure the stability of the study. CV was calculated by dividing the standard deviation by the arithmetic mean. If the coefficient of variation was less than 0.5, the round of Delphi investigation was terminated, and if it was more than 0.8, it was evaluated as unstable, and it was judged that an additional Delphi investigation was necessary [31].

Finally, this study evaluated the clarity of dental hygiene interventions and definitions. Clarity was evaluated on a five-point Likert scale through the question, “Is the content clearly written in understandable terms?”, as suggested by McCloskey and Bulechek [27]. The evaluation ranged from 1 point (not at all appropriate) to 5 points (very appropriate), and the content was judged to be clear when the average score was 4 points or more.

4. Research Streams for Dental Hygiene Intervention Classification

**Figure 1:**
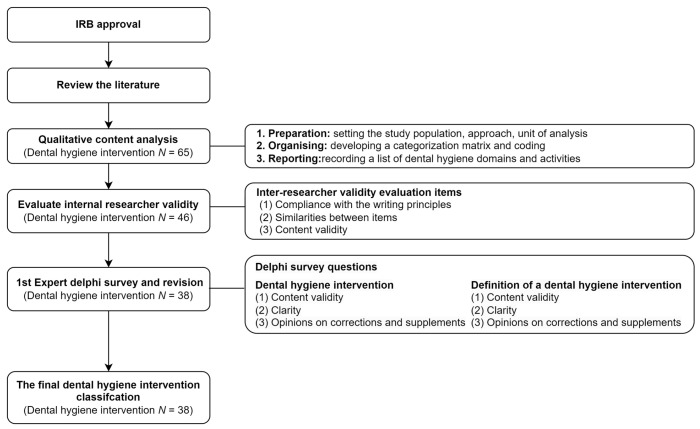
Study flow.

The study flow for the classification of dental hygiene interventions was presented in Figure 1.

## 3. Results

### 3.1. Results of the Expert Delphi Survey on Dental Hygiene Interventions

Table 2 shows the results of the expert Delphi survey on dental hygiene interventions. In the results of the content validity analysis of 46 dental hygiene interventions in the first Delphi survey on dental hygiene interventions, the average CVI was 0.87 or higher and CVR was 0.60 or higher. The degree of convergence was 0.50 in the items of ‘oral health screening, dental anxiety reduction, security enhancement, emotional support, pain management, and mutual goal setting, confirming that there are differences in scores between experts. The degree of agreement was 0.80 or higher, and the clarity was 4.40 or higher on average.

The first Delphi survey derived the final 38 dental hygiene interventions out of 46 by correcting and supplementing items with low convergence. In the results of the second Delphi survey on 38 dental hygiene interventions, the content validity of all 38 dental hygiene interventions was CVI 1.00 and CVR 1.00, respectively, and all expert opinions were evaluated as congruent. The clarity was evaluated as having an average score of 4.80 or higher, indicating that the dental hygiene intervention was clearly written in easy-to-understand terms.

### 3.2. Results of the Expert Delphi Survey on the Definition of Dental Hygiene Interventions

Table 3 shows the results of the expert Delphi survey regarding the definition of dental hygiene interventions. In the results of the first Delphi survey on the definition of dental hygiene interventions, the content validity of the definitions of 46 dental hygiene interventions showed an average CVI of 0.93 or higher and a CVR of 0.87 or higher. The degree of convergence was 0.50, in the definition of “oral health screening, security enhancement, and exchange of oral health information”, confirming that there was a difference in scores between experts. The degree of agreement was 0.80 or higher, and the clarity was 4.47 or higher on average.

In the results of conducting the second Delphi survey after revising and supplementing the definition of dental hygiene interventions according to expert opinion, the content validity for the definition of 38 dental hygiene interventions showed an average CVI of 0.93 or higher and CVR of 0.87 or higher. After correction and supplementation, the experts’ opinions on each definition were evaluated as congruent. In addition, clarity was evaluated with an average score of 4.87 or higher, and each definition was clearly written with contents that could explain dental hygiene interventions.

## 4. Discussion

This study conceptualized the distinctive practices offered by dental hygienists as dental hygiene interventions aimed at preventing oral diseases and enhancing oral health. To accomplish this, qualitative content analysis and expert Delphi surveys were conducted, drawing upon previous domestic and international studies. Consequently, 38 dental hygiene interventions were identified and conceptualized.

The dental hygiene intervention classification was a process of conceptualization by systematically grouping the unique practices of dental hygienists, which were previously listed independently according to similar characteristics, and naming using a generic term that could represent the essence of grouped practices [14]. Each dental hygiene intervention item named through the dental hygiene intervention classification was a concept that explains the professional behavior performed by dental hygienists and a dental hygiene term that could reveal the essential characteristics of dental hygiene and unique dental hygiene practice. Previous studies have explained methods for dental hygiene care, excluded practices related to behavior change, or focused on clinical techniques [5,6,11,13]. Accordingly, the meaning of the dental hygiene process of care performed by dental hygienists could not be sufficiently explained based on critical thinking, decision making, and professional judgment, and there were limitations in not revealing the expertise, originality, and comprehensiveness of dental hygiene practices. 

However, the dental hygiene intervention derived from this study has provided a clearer and more standardized understanding of dental hygienists’ unique practices. It was believed that this would not only elucidate the scope of their distinctive tasks but also serve as a foundation for establishing their expertise. Consequently, dental hygiene interventions and their definitions can effectively communicate the unique practices and expertise of dental hygienists to the public and other healthcare professionals, thereby raising awareness about the field of dental hygiene.

In addition, standardized terms established through the classification of dental hygiene interventions could be coded to facilitate documentation when recording the results of dental hygiene interventions. When information recorded in standardized codes was computerized into large-scale data, it would be possible to evaluate patient outcomes for interventions and to evaluate the contribution and effectiveness of interventions [32,33]. If a database recorded in standardized terms was built, it would be possible to analyze the cost-effectiveness of dental hygiene interventions, the effectiveness of cost savings, and the manpower and equipment required to perform the interventions [34]. To evaluate the effectiveness of dental hygiene interventions, provide grounds for performing interventions, and socially demonstrate the value of dental hygiene, it is necessary to establish a computerized program and system that can record standardized dental hygiene intervention terms. When establishing a computerized program, dental hygiene intervention items based on the dental hygiene process of care should be considered.

Dental hygiene intervention classification was the process of establishing the essence and attributes of dental hygiene practice and systematizing knowledge about dental hygiene [35]. Accordingly, we analyzed the classified dental hygiene interventions and identified five attributes of dental hygiene professionalism. First, it is thought that “dental hygiene care goal setting and plan”, “client’s informed consent”, “client rights protection”, and “education: dental hygiene care procedures” derived from this study were dental hygiene intervention items that conceptualized the activities of a dental hygienist who set dental hygiene care goals and priorities in cooperation with the clients, provided information on the dental hygiene process of care, and provided dental hygiene care with informed consent. Thus, it was considered that it reveals the essence of dental hygiene that pursues patient-centered oral care. Woodall [36] argued that it was important to focus on the needs of the client when applying dental hygiene care. She also emphasized the importance of actively involving the client when establishing goals and plans to conduct effective dental hygiene care. Therefore, it is crucial for dental hygienists to comprehend the individuality of each client, acknowledge their specific needs, and adopt an attitude that prioritizes the client as the primary agent in their treatment [37].

Second, “oral health risk identification, dental anxiety control, dental hygiene care goal setting and plan, client’s informed consent, education: dental hygiene care procedures, education: individual, and emotional support” derived from this study were dental hygiene intervention items that were performed to increase the degree of cooperation in the dental hygiene care of the client and to help the client control, decide, and change their health behavior based on therapeutic communication. These interventions contained the value of dental hygiene, which promotes active interaction of the dental hygienist with the client. To perform dental hygiene care, dental hygienists should be able to interact with the client in the process of oral health education and provide effective methods to promote the knowledge and behavioral change of the client as well as excellent clinical skills and knowledge [38]. A dental hygienist should involve the client as a co-therapist during dental hygiene care [2], collect accurate data through therapeutic communication, and then establish a treatment plan. The dental hygienist should be able to apply the optimal dental hygiene intervention through consultation and adjustment with the client [37]. When goals and plans for dental hygiene care are established through interaction with the client, the client might have a sense of purpose and could actively participate [39].

Third, among the dental hygiene intervention items derived from this study, “nutritional counseling”, “smoking cessation management”, “medication counseling”, “oral care product prescription”, “eating disorders and dysphagia care”, “dental caries management”, “periodontal disease management”, ”orthodontic patient care”, and “dental implant and prosthetic patient care” were dental hygiene intervention items that represented activities for a dental hygienist to provide comprehensive dental hygiene care based on the latest evidence to solve the client’s health problems. These interventions revealed the properties of the ‘evidence-based comprehensive approach’ aimed at dental hygiene science. To prevent oral diseases and promote the oral health of clients, comprehensive education and expert management that considers all aspects of socioeconomic, cognitive, emotional, and behavioral sciences, as well as clinical aspects, are required [10,37]. In particular, because oral health was significantly affected by health-related behaviors, in terms of behavioral science, the intervention to understand the behavior of the client and change the behavior is emphasized [40]. When providing such comprehensive care, evidence-based dental practices should be applied, taking into account the latest scientific evidence, the professionalism of the dental team, and sensitivity to the needs and preferences of the client [41].

Fourth, among the list of dental hygiene interventions derived from this study, “information exchange between health care providers, referral, support system enhancement” and “maintenance care” were dental hygiene interventions that represent activities to provide continuous management for the maintenance and promotion of oral health of the client. These interventions show the “continuity” of dental hygiene intervention based on the dental hygienist’s professionalism and responsibility for the client’s health. Particularly, due to the cyclical and ongoing nature of dental hygiene management, the health status and risk factors of the individual are consistently monitored, and continuous maintenance is provided [42]. Consequently, dental hygienists can offer essential information based on the client’s specific needs and promptly refer them to other dental providers if necessary. This ensures consistent and uninterrupted care delivery [43].

Fifth, the 38 dental hygiene interventions derived from this study reflected the paradigm of dental hygiene science that pursued the “prevention of oral diseases and promotion of oral health”. Dental hygiene science is the study of preventive oral healthcare, including the management of behaviors to prevent oral diseases and promote health [38]. The dental hygienist understood health and disease on a continuum and focused on improving the quality of life of the client based on the relationship between general health and oral health [44]. According to the paradigm of dental hygiene, dental hygiene science focused on health rather than disease, and by using the term “client” rather than “patient”, sought management for health promotion regardless of the presence or absence of disease. In addition, when performing dental hygiene care, it was emphasized that barriers to the health and oral health of the subject or socioeconomic, cultural, financial, and political environments should be considered [38,44].

As discussed above, dental hygienists could be defined as personnel who provide dental hygiene interventions based on the attributes of an evidence-based comprehensive approach, continuity, prevention of oral diseases, and pursuit of oral health promotion. Accordingly, the essence and professionalism of dental hygienists are reviewed to be similar to the concept of primary health care professionals defined by the World Health Organization and UNICEF. Prasad et al. [45] suggested that integrating oral health into primary health care is a demand of the times, and argued that primary oral health care needs to be developed as an integral part of primary health care. In particular, it was suggested that dental hygienists could be used for this purpose. In the future, by integrating oral health into the primary health care system and establishing a basic medical system in cooperation with multidisciplinary primary medical practitioners, it can contribute to improving general and oral health. It was required to actively utilize dental hygienists as primary oral health care professionals in the future to seek policy measures that could provide comprehensive and continuous preventive care to the public, and through this, it was expected that multidisciplinary primary health care providers would be able to contribute to the improvement of public health and oral health by establishing a primary medical system in cooperation.

The dental hygiene intervention classification derived from this study was limited in that it did not reflect the practice of dental hygienists active in the community because it focused on the clinical dental hygiene area. In addition, as the dental hygiene intervention classification was based on the scope of practices of some dental hygienists in Korea, it was difficult to generalize. Continuous discussions among professors, researchers, and clinicians are necessary to include the work of multidisciplinary and multinational dental hygienists in the future. Despite these limitations, this study was significant in that it conceptualized the unique practices of dental hygienists in Korea and prepared basic data necessary for expanding the body of knowledge in dental hygiene science.

## 5. Conclusions

As a result of this study, a total of 38 dental hygiene intervention items were derived through the dental hygiene intervention classification process based on qualitative content analysis. Each dental hygiene intervention item, identified during the classification process, represented a concept describing the interventions performed by dental hygienists. Simultaneously, these items served as dental hygiene terms, encapsulating the essential characteristics of dental hygiene science. Consequently, these 38 dental hygiene interventions can elucidate the professional activities of dental hygienists, rooted in critical thinking, decision making, and professional judgment. Moreover, this review underscores the potential for an increased understanding of the role and expertise of dental hygienists among other medical professionals and the public. The use of standardized dental hygiene intervention terms can contribute to the establishment of evidence-based dental hygiene science and the provision of high-quality dental hygiene care. Therefore, continuous efforts should be made within the academic community to achieve a global consensus on standardized terminology and curriculum, alongside conducting in-depth research in relevant fields. Additionally, it is recommended to engage in practical discussions to actively integrate dental hygienists into the primary care system, in addition to their role in dental clinical settings.

## Figures and Tables

**Table 1:** Expert Delphi survey assessment items.

Classification	Items	
Dental hygiene intervention	1.1.	Content validity: Is the corresponding intervention item appropriate as a ‘dental hygiene intervention’?
	1.2.	Clarity: Is the corresponding dental hygiene intervention written in clear and understandable terms?
	1.3.	Description of subjective opinions on corrections and supplements, etc.
Definition of a dental hygiene intervention	2.1.	Content validity: Is the definition of the corresponding dental hygiene intervention adequate?
	2.2.	Clarity: Is the definition clearly written to explain the dental hygiene intervention?
	2.3.	Description of subjective opinions on corrections and supplements, etc.

**Table 2:** Content validity of dental hygiene intervention labels.

First Delphi Survey										Second Delphi Survey								
No.	Dental Hygiene Intervention Labels	Content Validity						Clarity	Reflection ^‡^	No.	Dental Hygiene Intervention Labels	Content Validity						Clarity
		Mean ± SD	CVI ^†^	CVR ^†^	Convergence	Agreement	CV ^†^	Mean ± SD				Mean ± SD	CVI ^†^	CVR ^†^	Convergence	Agreement	CV ^†^	Mean ± SD
1	Oral health screening	4.53 ± 0.74	0.87	0.73	0.50	0.80	0.16	4.40 ± 0.91	C	1	Oral health risk identification	4.93 ± 0.26	1.00	1.00	0.00	1.00	0.05	4.80 ± 0.41
2	Dental anxiety reduction	4.53 ± 0.64	0.93	0.87	0.50	0.80	0.14	4.47 ± 0.74	C	2	Dental anxiety control	4.93 ± 0.26	1.00	1.00	0.00	1.00	0.05	4.93 ± 0.26
3	Security Enhancement	4.33 ± 1.11	0.80	0.60	0.50	0.80	0.26	4.67 ± 0.62	D		-	-	-	-	-	-	-	-
4	Emotional support	4.60 ± 0.63	0.93	0.87	0.50	0.80	0.14	4.67 ± 0.49	M	3	Emotional support	5.00 ± 0.00	1.00	1.00	0.00	1.00	0.00	5.00 ± 0.00
5	Pain management	4.67 ± 0.49	1.00	1.00	0.50	0.80	0.10	4.60 ± 0.63	D		-	-	-	-	-	-	-	-
6	Extraoral and intraoral assessment	4.93 ± 0.26	1.00	1.00	0.00	1.00	0.05	4.93 ± 0.26	M	4	Extraoral and intraoral assessment	5.00 ± 0.00	1.00	1.00	0.00	1.00	0.00	5.00 ± 0.00
7	Halitosis assessment	4.93 ± 0.26	1.00	1.00	0.00	1.00	0.05	5.00 ± 0.00	M	5	Halitosis assessment	5.00 ± 0.00	1.00	1.00	0.00	1.00	0.00	5.00 ± 0.00
8	Dentition and hard tissue assessment	4.87 ± 0.52	0.93	0.87	0.00	1.00	0.11	4.87 ± 0.52	M	6	Dentition and hard tissue assessment	5.00 ± 0.00	1.00	1.00	0.00	1.00	0.00	5.00 ± 0.00
9	Periodontal assessment	5.00 ± 0.00	1.00	1.00	0.00	1.00	0.00	5.00 ± 0.00	M	7	Periodontal assessment	5.00 ± 0.00	1.00	1.00	0.00	1.00	0.00	5.00 ± 0.00
10	Oral hygiene assessment	4.93 ± 0.26	1.00	1.00	0.00	1.00	0.05	4.93 ± 0.26	M	8	Oral hygiene assessment	5.00 ± 0.00	1.00	1.00	0.00	1.00	0.00	5.00 ± 0.00
11	Dental radiography	5.00 ± 0.00	1.00	1.00	0.00	1.00	0.00	5.00 ± 0.00	M	9	Dental radiography	5.00 ± 0.00	1.00	1.00	0.00	1.00	0.00	5.00 ± 0.00
12	Oral health problems diagnosis	4.93 ± 0.26	1.00	1.00	0.00	1.00	0.05	4.93 ± 0.26	M	10	Oral health problems diagnosis	4.93 ± 0.26	1.00	1.00	0.00	1.00	0.05	5.00 ± 0.00
13	Mutual goal setting	4.53 ± 0.74	0.87	0.73	0.50	0.80	0.16	4.40 ± 0.63	C	11	Dental hygiene care goal setting and plan	4.87 ± 0.35	1.00	1.00	0.00	1.00	0.07	4.80 ± 0.41
14	Client’s informed consent	4.93 ± 0.26	1.00	1.00	0.00	1.00	0.05	4.87 ± 0.35	M	12	Client’s informed consent	5.00 ± 0.00	1.00	1.00	0.00	1.00	0.00	5.00 ± 0.00
15	Oral health care information exchange	4.60 ± 0.74	0.87	0.73	0.25	0.90	0.16	4.47 ± 0.83	C	13	Information exchange between healthcare providers	4.80 ± 0.41	1.00	1.00	0.00	1.00	0.09	4.80 ± 0.41
16	Referral	4.73 ± 0.59	0.93	0.87	0.00	1.00	0.13	4.73 ± 0.59	M	14	Referral	4.93 ± 0.26	1.00	1.00	0.00	1.00	0.05	4.87 ± 0.35
17	Dental equipment management	4.60 ± 0.74	0.87	0.73	0.25	0.90	0.16	4.60 ± 0.74	D		-	-	-	-	-	-	-	-
18	Staff development	4.60 ± 0.74	0.87	0.73	0.25	0.90	0.16	4.73 ± 0.59	D		-	-	-	-	-	-	-	-
19	Health insurance management	4.73 ± 0.59	0.93	0.87	0.00	1.00	0.13	4.73 ± 0.46	D		-	-	-	-	-	-	-	-
20	Infection control	5.00 ± 0.00	1.00	1.00	0.00	1.00	0.00	4.93 ± 0.26	M	15	Infection control	5.00 ± 0.00	1.00	1.00	0.00	1.00	0.00	5.00 ± 0.00
21	Client rights protection	4.73 ± 0.80	0.93	0.87	0.00	1.00	0.17	4.93 ± 0.26	M	16	Client rights protection	4.93 ± 0.26	1.00	1.00	0.00	1.00	0.05	4.93 ± 0.26
22	Education: dental hygiene care procedures	5.00 ± 0.00	1.00	1.00	0.00	1.00	0.00	4.93 ± 0.26	M	17	Education: dental hygiene care procedures	5.00 ± 0.00	1.00	1.00	0.00	1.00	0.00	5.00 ± 0.00
23	Education: precautions before and after surgery	4.73 ± 0.80	0.93	0.87	0.00	1.00	0.17	4.87 ± 0.35	D		-	-	-	-	-	-	-	-
24	Education: individual	5.00 ± 0.00	1.00	1.00	0.00	1.00	0.00	4.87 ± 0.35	M	18	Education: individual	5.00 ± 0.00	1.00	1.00	0.00	1.00	0.00	5.00 ± 0.00
25	Education: life cycle	5.00 ± 0.00	1.00	1.00	0.00	1.00	0.00	5.00 ± 0.00	D		-	-	-	-	-	-	-	-
26	Education: patients with systemic diseases	5.00 ± 0.00	1.00	1.00	0.00	1.00	0.00	4.93 ± 0.26	D		-	-	-	-	-	-	-	-
27	Education: special patients	5.00 ± 0.00	1.00	1.00	0.00	1.00	0.00	5.00 ± 0.00	C	19	Oral health care for special patients	5.00 ± 0.00	1.00	1.00	0.00	1.00	0.00	5.00 ± 0.00
28	Education: nutritional counseling	4.93 ± 0.26	1.00	1.00	0.00	1.00	0.05	5.00 ± 0.00	C	20	Nutritional counseling	5.00 ± 0.00	1.00	1.00	0.00	1.00	0.00	5.00 ± 0.00
29	Education: smoking cessation	4.93 ± 0.26	1.00	1.00	0.00	1.00	0.05	5.00 ± 0.00	C	21	Smoking cessation management	4.93 ± 0.26	1.00	1.00	0.00	1.00	0.05	5.00 ± 0.00
30	Oral care products recommendation	4.93 ± 0.26	1.00	1.00	0.00	1.00	0.05	5.00 ± 0.00	M	22	Oral care products prescription	5.00 ± 0.00	1.00	1.00	0.00	1.00	0.00	5.00 ± 0.00
31	Eating disorders patients care	4.87 ± 0.35	1.00	1.00	0.00	1.00	0.07	4.93 ± 0.26	C	23	Eating disorders and dysphagia care	5.00 ± 0.00	1.00	1.00	0.00	1.00	0.00	5.00 ± 0.00
32	Dry mouth care	5.00 ± 0.00	1.00	1.00	0.00	1.00	0.00	5.00 ± 0.00	M	24	Dry mouth management	5.00 ± 0.00	1.00	1.00	0.00	1.00	0.00	5.00 ± 0.00
33	Dentinal hypersensitivity control	5.00 ± 0.00	1.00	1.00	0.00	1.00	0.00	5.00 ± 0.00	M	25	Dentinal hypersensitivity control	5.00 ± 0.00	1.00	1.00	0.00	1.00	0.00	5.00 ± 0.00
34	Halitosis management	4.93 ± 0.26	1.00	1.00	0.00	1.00	0.05	5.00 ± 0.00	M	26	Halitosis management	5.00 ± 0.00	1.00	1.00	0.00	1.00	0.00	5.00 ± 0.00
35	Prosthetic treatment management	5.00 ± 0.00	1.00	1.00	0.00	1.00	0.00	4.93 ± 0.26	C	27	Dental implant and prosthetic patient care	5.00 ± 0.00	1.00	1.00	0.00	1.00	0.00	5.00 ± 0.00
36	Dental caries management	5.00 ± 0.00	1.00	1.00	0.00	1.00	0.00	5.00 ± 0.00	M	28	Dental caries management	5.00 ± 0.00	1.00	1.00	0.00	1.00	0.00	5.00 ± 0.00
37	Periodontal disease management	5.00 ± 0.00	1.00	1.00	0.00	1.00	0.00	5.00 ± 0.00	M	29	Periodontal disease management	5.00 ± 0.00	1.00	1.00	0.00	1.00	0.00	5.00 ± 0.00
38	Orthodontic patient management	4.93 ± 0.26	1.00	1.00	0.00	1.00	0.05	4.93 ± 0.26	C	30	Orthodontic patient care	5.00 ± 0.00	1.00	1.00	0.00	1.00	0.00	5.00 ± 0.00
39	Client behavioral control	4.93 ± 0.26	1.00	1.00	0.00	1.00	0.05	5.00 ± 0.00	M	31	Client behavioral control	5.00 ± 0.00	1.00	1.00	0.00	1.00	0.00	5.00 ± 0.00
40	Dental emergency care	4.93 ± 0.26	1.00	1.00	0.00	1.00	0.05	5.00 ± 0.00	M	32	Dental emergency care	5.00 ± 0.00	1.00	1.00	0.00	1.00	0.00	5.00 ± 0.00
41	Support system enhancement	4.73 ± 0.59	0.93	0.87	0.00	1.00	0.13	4.80 ± 0.41	M	33	Support system enhancement	4.93 ± 0.26	1.00	1.00	0.00	1.00	0.05	4.93 ± 0.26
42	Education: Medication counseling before and after treatment	4.87 ± 0.52	0.93	0.87	0.00	1.00	0.11	5.00 ± 0.00	C	34	Medication counseling	5.00 ± 0.00	1.00	1.00	0.00	1.00	0.00	5.00 ± 0.00
43	Dental hygiene care evaluation	5.00 ± 0.00	1.00	1.00	0.00	1.00	0.00	5.00 ± 0.00	M	35	Dental hygiene care evaluation	5.00 ± 0.00	1.00	1.00	0.00	1.00	0.00	5.00 ± 0.00
44	Satisfaction survey	4.67 ± 0.62	0.93	0.87	0.25	0.90	0.13	4.73 ± 0.46	C	36	Satisfaction evaluation	4.93 ± 0.26	1.00	1.00	0.00	1.00	0.05	4.93 ± 0.26
45	Maintenance care	5.00 ± 0.00	1.00	1.00	0.00	1.00	0.00	5.00 ± 0.00	M	37	Maintenance care	5.00 ± 0.00	1.00	1.00	0.00	1.00	0.00	5.00 ± 0.00
46	Dental hygiene documentation	5.00 ± 0.00	1.00	1.00	0.00	1.00	0.00	5.00 ± 0.00	M	38	Dental hygiene documentation	5.00 ± 0.00	1.00	1.00	0.00	1.00	0.00	5.00 ± 0.00

^†^ CVI: content validity index, CVR: content validity ratio, CV: coefficient of variation. ^‡^ C: correction, D: Deletion, M: maintenance.

**Table 3:** Content validity of the definition of a dental hygiene intervention.

First Delphi Survey											Second Delphi Survey									
No.	Dental Hygiene Intervention Labels	Definition	Content Validity						Clarity	Reflection ^‡^	No.	Dental Hygiene Intervention Labels	Definition	Content Validity					Clarity	
			Mean ± SD	CVI ^†^	CVR ^†^	Convergence	Agreement	CV ^†^	Mean ± SD					Mean ± SD	CVI ^†^	CVR ^†^	Convergence	Agreement	CV ^†^	Mean ± SD
1	Oral health screening	Assessing potential oral health risk factors or problems	4.67 ± 0.49	1.00	1.00	0.50	0.80	0.10	4.47 ± 0.64	M	1	Oral health risk identification	Assessing potential oral health risk factors or problems	4.98 ± 0.09	1.00	1.00	0.00	1.00	0.02	4.90 ± 0.28
2	Dental anxiety reduction	Measuring the subjective level of dental anxiety of the client and helping the client control their anxiety response	4.73 ± 0.46	1.00	1.00	0.25	0.90	0.10	4.80 ± 0.41	M	2	Dental anxiety control	Measuring the subjective level of dental anxiety of the client and helping the client control their anxiety response	4.93 ± 0.26	1.00	1.00	0.00	1.00	0.05	4.87 ± 0.35
3	Security Enhancement	Strengthening the client’s physical and psychological stability before, during, and after dental hygiene care	4.60 ± 0.51	1.00	1.00	0.50	0.80	0.11	4.67 ± 0.49	D		-		-	-	-	-	-	-	-
4	Emotional support	Provision of reassurance, acceptance, and encouragement during times of stress	4.73 ± 0.46	1.00	1.00	0.25	0.90	0.10	4.67 ± 0.49	M	3	Emotional support	Provision of reassurance, acceptance, and encouragement during times of stress	5.00 ± 0.00	1.00	1.00	0.00	1.00	0.00	5.00 ± 0.00
5	Pain management	Identifying the factors in the patient’s pain response and helping them control the pain	4.80 ± 0.41	1.00	1.00	0.00	1.00	0.09	4.67 ± 0.62	D		-		-	-	-	-	-	-	-
6	Extraoral and intraoral assessment	Detecting abnormal conditions and parts suspected of disease through palpation and observation the inside and outside of the mouth	4.80 ± 0.41	1.00	1.00	0.00	1.00	0.09	4.73 ± 0.59	C	4	Extraoral and intraoral assessment	Inspecting and palpating the extra-oral and intra-oral to detect the abnormal or suspicious status of the disease	5.00 ± 0.00	1.00	1.00	0.00	1.00	0.00	5.00 ± 0.00
7	Halitosis assessment	Identifying the degree of expiratory gas that client or the other finds unpleasant and related factors	4.87 ± 0.35	1.00	1.00	0.00	1.00	0.07	4.73 ± 0.70	C	5	Halitosis assessment	Assessing risk factors associated with halitosis that the client or others find unpleasant	5.00 ± 0.00	1.00	1.00	0.00	1.00	0.00	4.87 ± 0.35
8	Dentition and hard tissue assessment	Assessing teeth and hard tissues to detect the abnormal or suspicious status of the disease	4.93 ± 0.26	1.00	1.00	0.00	1.00	0.05	4.93 ± 0.26	M	6	Dentition and hard tissue assessment	Assessing teeth and hard tissues to detect the abnormal or suspicious status of the disease	5.00 ± 0.00	1.00	1.00	0.00	1.00	0.00	5.00 ± 0.00
9	Periodontal assessment	Assessing periodontal tissue to detect the abnormal or suspicious status of the disease	4.87 ± 0.52	0.93	0.87	0.00	1.00	0.11	5.00 ± 0.00	M	7	Periodontal assessment	Assessing periodontal tissue to detect the abnormal or suspicious status of the disease	5.00 ± 0.00	1.00	1.00	0.00	1.00	0.00	5.00 ± 0.00
10	Oral hygiene assessment	Detecting soft and hard attachments	4.87 ± 0.52	0.93	0.87	0.00	1.00	0.11	4.87 ± 0.35	C	8	Oral hygiene assessment	Assessing soft and hard attachments in the oral cavity	4.93 ± 0.26	1.00	1.00	0.00	1.00	0.05	5.00 ± 0.00
11	Dental radiography	Taking a dental radiograph and checking the extent, location, and progress of the lesion	4.93 ± 0.26	1.00	1.00	0.00	1.00	0.05	5.00 ± 0.00	M	9	Dental radiography	Taking a dental radiograph and checking the extent, location, and progress of the lesion	4.93 ± 0.26	1.00	1.00	0.00	1.00	0.05	5.00 ± 0.00
12	Oral health problems diagnosis	Determining a client‘s unmet dental hygiene’s human needs and oral health problems based on potential risk factors	4.80 ± 0.56	0.93	0.87	0.00	1.00	0.12	4.87 ± 0.52	M	10	Oral health problems diagnosis	Determining a client‘s unmet dental hygiene’s human needs and oral health problems based on potential risk factors	5.00 ± 0.00	1.00	1.00	0.00	1.00	0.00	5.00 ± 0.00
13	Mutual goal setting	Developing a plan by determining dental hygiene management goals and priorities in cooperation with the client	4.73 ± 0.59	0.93	0.87	0.00	1.00	0.13	4.73 ± 0.46	C	11	Dental hygiene care goal setting and plan	Collaborating with client to identify and prioritize dental hygiene care goals, then developing a plan for achieving those goals	5.00 ± 0.00	1.00	1.00	0.00	1.00	0.00	4.93 ± 0.26
14	Client’s informed consent	Providing necessary information to make decisions on dental hygiene care and obtaining consent from the client for a dental hygiene care plan	4.93 ± 0.26	1.00	1.00	0.00	1.00	0.05	4.87 ± 0.52	C	12	Client’s informed consent	Providing the client with information on dental hygiene care and obtaining consent from the client for care plan and implementation	5.00 ± 0.00	1.00	1.00	0.00	1.00	0.00	5.00 ± 0.00
15	Oral health care information exchange	Strengthening collaboration among multidisciplinary health care providers and providing information about clients	4.60 ± 0.63	0.93	0.87	0.50	0.80	0.14	4.60 ± 0.74	C	13	Information exchange between healthcare providers	Providing information about the client to strengthen cooperation between healthcare providers	4.87 ± 0.35	1.00	1.00	0.00	1.00	0.07	4.93 ± 0.26
16	Referral	Providing information about the client and linking them to medical services to other health professionals	4.87 ± 0.35	1.00	1.00	0.00	1.00	0.07	4.73 ± 0.59	C	14	Referral	Providing information about the client, then arranging for services by another care provider or agency	4.93 ± 0.26	1.00	1.00	0.00	1.00	0.05	5.00 ± 0.00
17	Dental equipment management	Maintenance and management of dental equipment and consumables for dental treatment	4.67 ± 0.62	0.93	0.87	0.25	0.90	0.13	4.67 ± 0.49	D		-		-	-	-	-	-	-	-
18	Staff development	Developing, maintaining, and monitoring employee competencies	4.67 ± 0.82	0.93	0.87	0.00	1.00	0.17	4.60 ± 0.83	D		-		-	-	-	-	-	-	-
19	Health insurance management	Performing health insurance claims management to secure payment for health services	4.87 ± 0.35	1.00	1.00	0.00	1.00	0.07	4.93 ± 0.26	D		-		-	-	-	-	-	-	-
20	Infection control	Minimize the possibility and transmission of infection by reducing or eliminating microorganisms that may be shared between individuals or between individuals and contaminated surfaces	4.87 ± 0.52	0.93	0.87	0.00	1.00	0.11	4.87 ± 0.52	C	15	Infection control	Minimizing the possibility of infection and spread to prevent the spread of infectious agents	4.87 ± 0.52	0.93	0.87	0.00	1.00	0.11	4.87 ± 0.52
21	Client rights protection	Protection of health care rights of a client	5.00 ± 0.00	1.00	1.00	0.00	1.00	0.00	5.00 ± 0.00	M	16	Client rights protection	Protection of health care rights of a client	5.00 ± 0.00	1.00	1.00	0.00	1.00	0.00	5.00 ± 0.00
22	Education: Dental hygiene care procedures	Helping the client understand and prepare for dental hygiene care procedure and treatment	5.00 ± 0.00	1.00	1.00	0.00	1.00	0.00	5.00 ± 0.00	C	17	Education: Dental Hygiene care procedures	Providing information to the client so that they understand and prepare for dental hygiene care procedures or treatment.	5.00 ± 0.00	1.00	1.00	0.00	1.00	0.00	5.00 ± 0.00
23	Education: precautions before and after surgery	Helping the client to understand the requirements for recovery and maintenance of the client before and after surgery	4.87 ± 0.52	0.93	0.87	0.00	1.00	0.11	4.87 ± 0.52	D		-		-	-	-	-	-	-	-
24	Education: Individual	Improving oral hygiene and promoting oral health	4.80 ± 0.56	0.93	0.87	0.00	1.00	0.12	4.73 ± 0.70	M	18	Education: Individual	Improving oral hygiene and promoting oral health	5.00 ± 0.00	1.00	1.00	0.00	1.00	0.00	4.93 ± 0.26
25	Education: life cycle	Improving the oral health of clients by age of life cycle	5.00 ± 0.00	1.00	1.00	0.00	1.00	0.00	5.00 ± 0.00	D		-								
26	Education: patients with systemic diseases	Improving the oral health of clients with systemic diseases	4.87 ± 0.52	0.93	0.87	0.00	1.00	0.11	4.87 ± 0.35	D		-								
27	Education: special patients	Improving the oral health of special patients, including bedridden patients, the disabled, cleft lip and palate patients, etc.	4.87 ± 0.35	1.00	1.00	0.00	1.00	0.07	4.80 ± 0.41	C	19	Oral health care for special patients	Promoting the oral health of special patients (systemic diseases, geriatric diseases (bed sick, cancer patients, dementia patients, stroke patients), disabled people, etc.)	5.00 ± 0.00	1.00	1.00	0.00	1.00	0.00	4.93 ± 0.26
28	Education: nutritional counseling	Helping with dietary adjustments to improve oral health	4.93 ± 0.26	1.00	1.00	0.00	1.00	0.05	4.93 ± 0.26	C	20	Nutritional counseling	Managing diet to promote oral health	5.00 ± 0.00	1.00	1.00	0.00	1.00	0.00	5.00 ± 0.00
29	Education: smoking cessation	Motivating and helping the client to stop smoking and use nicotine	4.93 ± 0.26	1.00	1.00	0.00	1.00	0.05	4.93 ± 0.26	M	21	Smoking cessation management	Motivating and helping the client to stop smoking and nicotine	5.00 ± 0.00	1.00	1.00	0.00	1.00	0.00	5.00 ± 0.00
30	Oral care products prescription	Prescribing suitable oral care products for the client to prevent oral disease	5.00 ± 0.00	1.00	1.00	0.00	1.00	0.00	5.00 ± 0.00	C	22	Oral care products recommendation	Recommendation of suitable oral care products to prevent oral disease	5.00 ± 0.00	1.00	1.00	0.00	1.00	0.00	5.00 ± 0.00
31	Eating disorders patients care	Helping to recover oral function so that the client has no problem with eating or swallowing	4.93 ± 0.26	1.00	1.00	0.00	1.00	0.05	4.93 ± 0.26	C	23	Eating disorders and dysphagia care	Providing care to restore oral function so that the client has no problems with eating and swallowing	5.00 ± 0.00	1.00	1.00	0.00	1.00	0.00	4.93 ± 0.26
32	Dry mouth care	Identifying and relieving dry mouth	4.93 ± 0.26	1.00	1.00	0.00	1.00	0.05	4.87 ± 0.35	C	24	Dry mouth management	Assessing and relieving dry mouth	5.00 ± 0.00	1.00	1.00	0.00	1.00	0.00	4.93 ± 0.26
33	Dentinal hypersensitivity control	Identifying and relieving dentinal hypersensitivity	4.93 ± 0.26	1.00	1.00	0.00	1.00	0.05	4.87 ± 0.35	C	25	Dentinal hypersensitivity control	Assessing and relieving dentinal hypersensitivity	5.00 ± 0.00	1.00	1.00	0.00	1.00	0.00	5.00 ± 0.00
34	Halitosis management	Identifying and managing unpleasant halitosis	4.87 ± 0.35	1.00	1.00	0.00	1.00	0.07	4.93 ± 0.26	C	26	Halitosis management	Management to relieve unpleasant halitosis	5.00 ± 0.00	1.00	1.00	0.00	1.00	0.00	5.00 ± 0.00
35	Prosthetic treatment management	Healing and enhancing the periodontal tissue around the prosthesis before and after prosthetic treatment	4.93 ± 0.26	1.00	1.00	0.00	1.00	0.05	4.93 ± 0.26	C	27	Dental implant and prosthetic patient care	Management of improving the periodontal tissue and periodontal health around the prosthesis before and after dental implant and prosthetic treatment	4.93 ± 0.26	1.00	1.00	0.00	1.00	0.05	4.93 ± 0.26
36	Dental caries management	Preventing dental caries and improving dental status by controlling dental caries risk factors	5.00 ± 0.00	1.00	1.00	0.00	1.00	0.00	4.80 ± 0.41	C	28	Dental caries management	Preventing dental caries and improving dental health by controlling dental caries risk factors	5.00 ± 0.00	1.00	1.00	0.00	1.00	0.00	4.93 ± 0.26
37	Periodontal disease management	Preventing periodontal disease and improving periodontal status by controlling periodontal disease risk factors	5.00 ± 0.00	1.00	1.00	0.00	1.00	0.00	4.87 ± 0.35	C	29	Periodontal disease management	Preventing periodontal disease and improving periodontal health by controlling periodontal disease risk factors	5.00 ± 0.00	1.00	1.00	0.00	1.00	0.00	5.00 ± 0.00
38	Orthodontic patient management	Improving the oral health of patients undergoing orthodontic treatment	5.00 ± 0.00	1.00	1.00	0.00	1.00	0.00	4.93 ± 0.26	M	30	Orthodontic patient care	Improving the oral health of patients undergoing orthodontic treatment	5.00 ± 0.00	1.00	1.00	0.00	1.00	0.00	5.00 ± 0.00
39	Client behavioral control	Controlling behavioral patterns of uncooperative and aggressive clients during dental treatment and care	4.93 ± 0.26	1.00	1.00	0.00	1.00	0.05	4.93 ± 0.26	C	31	Client behavioral control	Controlling behavioral patterns of uncooperative clients during clinical treatment and care	5.00 ± 0.00	1.00	1.00	0.00	1.00	0.00	5.00 ± 0.00
40	Dental emergency care	Providing evaluation and measures for urgent dental situations	4.93 ± 0.26	1.00	1.00	0.00	1.00	0.05	4.93 ± 0.26	M	32	Dental emergency care	Providing evaluation and measures for dental urgent situations	5.00 ± 0.00	1.00	1.00	0.00	1.00	0.00	5.00 ± 0.00
41	Support system enhancement	Facilitation of support to patients by family, friends, and community	4.80 ± 0.41	1.00	1.00	0.00	1.00	0.09	4.73 ± 0.46	M	33	Support system enhancement	Facilitation of support to patients by family, friends, and community	5.00 ± 0.00	1.00	1.00	0.00	1.00	0.00	5.00 ± 0.00
42	Education: Medication counseling before and after treatment	Promoting the safe and efficient use of drugs, including prescription drugs and over-the-counter drugs	5.00 ± 0.00	1.00	1.00	0.00	1.00	0.00	4.93 ± 0.26	C	34	Medication counseling	Assisting the safe and efficient use of drugs, including prescription drugs and over-the-counter drugs	5.00 ± 0.00	1.00	1.00	0.00	1.00	0.00	5.00 ± 0.00
43	Dental hygiene care evaluation	Evaluating the client’s dental hygiene’s human needs and dental hygiene care goals	5.00 ± 0.00	1.00	1.00	0.00	1.00	0.00	5.00 ± 0.00	M	35	Dental hygiene care evaluation	Evaluating the client‘s dental hygiene’s human needs and dental hygiene care goals achievement	5.00 ± 0.00	1.00	1.00	0.00	1.00	0.00	5.00 ± 0.00
44	Satisfaction survey	Evaluating the client’s or guardian’s opinion about the dental hygiene management course service	4.87 ± 0.35	1.00	1.00	0.00	1.00	0.07	4.80 ± 0.41	C	36	satisfaction evaluation	Evaluating the client’s or guardian’s opinion on the dental hygiene process of care and giving feedback	4.93 ± 0.26	1.00	1.00	0.00	1.00	0.05	5.00 ± 0.00
45	Maintenance care	Assisting in maintaining the oral health status achieved by dental hygiene interventions	5.00 ± 0.00	1.00	1.00	0.00	1.00	0.00	5.00 ± 0.00	M	37	Maintenance care	Assisting in maintaining the oral health status achieved by dental hygiene interventions	5.00 ± 0.00	1.00	1.00	0.00	1.00	0.00	5.00 ± 0.00
46	Dental hygiene documentation	Recording important data about the client in the dental hygiene care record	5.00 ± 0.00	1.00	1.00	0.00	1.00	0.00	5.00 ± 0.00	M	38	Dental hygiene documentation	Recording important data about the client in the dental hygiene care record	5.00 ± 0.00	1.00	1.00	0.00	1.00	0.00	5.00 ± 0.00

^†^ CVI: content validity index, CVR: content validity ratio, CV: coefficient of variation. ^‡^ C: correction, D: Deletion, M: maintenance.

## Data Availability

The data presented in this study are available in within the article.
